# An Overview of the Use of Anti-Angiogenic Agents in the Treatment of Thymic Epithelial Tumors

**DOI:** 10.3390/ijms242317065

**Published:** 2023-12-02

**Authors:** Apostolos C. Agrafiotis, Lawek Berzenji, Stien Koyen, Dries Vermeulen, Rachel Winthagen, Jeroen M. H. Hendriks, Paul E. Van Schil

**Affiliations:** 1 Department of Thoracic and Vascular Surgery, Antwerp University Hospital, B-2650 Edegem, Belgium; 2 Department of Thoracic and Vascular Surgery, Wallonie Picarde Hospital Center (Centre Hospitalier de Wallonie Picarde—CHwapi), B-7500 Tournai, Belgium; 3 ASTARC, University of Antwerp, B-2610 Wilrijk, Belgium

**Keywords:** angiogenesis, thymic epithelial tumors, thymoma, thymic carcinoma

## Abstract

Angiogenesis significantly influences the carcinogenesis of thymic epithelial tumors (TET). Both thymomas and thymic carcinoma (TC) overexpress VEGF-A and VEGFR-1 and -2. This review aims to provide an appraisal of the use of anti-angiogenics in the treatment of TET. The literature research identified 16 studies that were deemed eligible for further analysis. Seven studies assessed the clinical efficacy of sunitinib and five studies the use of apatinib and/or anlotinib. The multicenter Japanese phase II REMORA trial investigated the efficacy of lenvatinib, which is a multi-targeted inhibitor of VEGFR, FGFR, RET, c-Kit, and other kinases. The objective response rate was 38% (25.6–52%), which is the highest documented in TET that progressed after first-line chemotherapy. Anti-angiogenic agents may be useful in the treatment of TET, which are not amenable to curative treatment. Their toxicity profile seems to be acceptable. However, angiogenesis inhibitors do not appear to have a major influence on either thymomas or TC, although multikinase inhibitors may have some effect on TC. The current evidence suggests that the most active agent is lenvatinib, whereas sunitinib could be proposed as an acceptable second-line therapy for TC. Further research concerning the combination of immune checkpoint inhibitors with anti-angiogenic drugs is warranted.

## 1. Introduction

The creation of new blood vessels is known as angiogenesis [1]. Endothelial cells, which line the interior of blood arteries, move, proliferate, and differentiate during this process [2].

Chemical messengers in the body control the angiogenesis process. Some of these signals, such as vascular endothelial growth factor (VEGF), attach to receptors on the surface of healthy endothelial cells [3]. Signals within endothelial cells are elicited when VEGF and other endothelial growth factors attach to their receptors on these cells, promoting the creation and survival of new blood vessels [1]. A different class of chemical signals known as angiogenesis inhibitors prevents the growth of blood vessels. In general, these chemical signals’ effects on angiogenesis are balanced between their stimulatory and inhibitory actions [4]. 

Solid tumors require a blood supply in order to grow larger; hence, angiogenesis is crucial to the development of cancer [1,5]. Actually, tumors can induce angiogenesis by emitting chemical signals that cause it to occur [6]. Additionally, tumors can induce the production of angiogenesis signaling molecules in surrounding normal cells [4].

The newly formed blood vessels provide oxygen and other necessary elements to the expanding tumors, enabling them to grow larger and provide the ability to the cancer cells to penetrate neighboring tissue, spread throughout the body, and generate metastases [1,5,6]. Without a blood supply, cancer colonies cannot grow or spread past a certain size; thus, researchers have created drugs called angiogenesis inhibitors that intervene with tumor angiogenesis [7]. These anti-angiogenic agents aim to stop or limit the growth of cancer by depriving it of the blood supply it requires. Instead of preventing the growth of tumor cells, these medications prevent the growth of blood vessels that are required for tumor enlargement.

Angiogenesis inhibitors disrupt different phases of blood vessel formation in a number of different ways. Some are VEGF-specific monoclonal antibodies that bind to VEGF [7,8]. VEGF is unable to activate the VEGF receptor when bound to these agents. Other inhibitors of angiogenesis bind to VEGF and/or its receptor as well as to additional receptors found on the surface of endothelial cells or to additional proteins in the downstream signaling cascades, preventing their activities [7]. Some immunomodulatory agents (substances that boost or decrease the immune system) also have anti-angiogenic characteristics. Most solid tumors have elevated levels of VEGF, which is commonly regarded as a critical mediator of tumor angiogenesis. Due to this, VEGF/VEGFR inhibitor development has dominated the development of anti-angiogenics during the past few decades [8].

Tumor blood vessels differ greatly from normal blood vessels and are not genetically unstable, making them a potentially important target for cancer treatment [9]. For a tumor to begin neovascularization, it needs to transform into an angiogenic phenotype. While genetics may play a part, sensitivity to environmental stressors, such as hypoxia, is also important. Evidence implies that the acquisition of this characteristic occurs early in tumor formation and that it is rate-limiting for tumor progression. The majority of the evidence comes from transgenic models with repeatable histologically different tumor phases. Strong angiogenic phenotypes are observed in cells transformed by RAS, MYC, RAF, c-erbB-2, c-JUN, or SRC. This is partially due to overexpression of vascular endothelial growth factor (VEGF) or reciprocal downregulation of thrombospondin 1 [10,11,12,13,14,15]. 

Furthermore, although it has been observed that mutant P53 increases VEGF, wild-type P53 may simultaneously reduce VEGF synthesis and raise the levels of angiogenesis suppressor thrombospondin 1. These routes result in the formation of aberrant, leaky vasculature with blind sacs, reversed, and intermittent flow. As a result, medication and oxygen delivery is significantly worse than in normal tissues despite an increase in the development of new arteries. The genetic selection of cancer cells and their resistance to drugs and radiation are both influenced by this complicated environment [9].

Thymomas and thymic carcinomas are included in the rare group of cancers of the anterior mediastinum known as thymic epithelial tumors (TET). Their origin is the thymus’ epithelial cells. In fact, the most common tumor in the prevascular mediastinum is thymoma. Thymic carcinoma is estimated to rate at about 15% to 20% of all thymic neoplasms. Although some of these infrequent neoplasms were long thought to be benign lesions, because of their indolent course, they are now recognized as malignant neoplasms with the potential to occasionally be highly aggressive and metastatic. Their yearly incidence is 0.15 cases per 100,000. Specifically, thymic carcinoma is a very unusual tumor that spreads to neighboring tissues. As a result, its 5-year survival rate is between 30% and 50% lower than that of thymomas [16].

Anti-angiogenics have shown their potential efficacy in many solid tumors; however, their place in the treatment of thymic epithelial tumors (TET) comprising thymoma and thymic carcinoma (TC) is less clear [4]. Angiogenesis significantly influences TET carcinogenesis. Both thymomas and TC overexpress VEGF-A and VEGFR-1 and -2, although there is little information on the effectiveness of angiogenesis inhibitors in thymic malignancies [3]. Low response rates were observed with bevacizumab [17]. On the contrary, the activity of multikinase inhibitors, such as sorafenib and sunitinib, has been emphasized in case reports involving TC [18,19].

This review aims to provide an appraisal of the current evidence regarding the use of anti-angiogenics in the treatment of thymomas and TC.

## 2. Results

The literature research identified sixteen studies that were deemed eligible for further analysis [17,18,19,20,21,22,23,24,25,26,27,28,29,30,31,32,33,34]. There were eight phase II trials, four retrospective series, one phase I study, one prospective series, and two case reports. Seven studies assessed the clinical efficacy of sunitinib and five studies the use of apatinib and/or anlotinib. The phase II trials with published results and the prospective series are presented in Table 1. 

The phase II Resound trial enrolled nineteen patients (eight male/eleven female) with TET who received regorafenib, a VEGFR–PDGFR–FGFR inhibitor [20]. There were six B2 thymomas, five B3 thymomas, and eight TC that were previously treated with a platinum-based chemotherapy scheme showing progressive disease (PD). The median follow-up (mFU) was 39.1 months and the primary endpoint was the 8-week progression-free survival (PFS) rate. The median PFS was 9.6 months (95% CI, 3.6–12.8) and the median overall survival (mOS) was 33.8 months (95% CI, 10.2 not reached). Grade 3–4 treatment-related adverse events (AE) were observed in 52.6% of cases, the most frequent being hypertension (10.5%) and lipase elevation (5.3%) [20].

A multicenter prospective cohort assessed the efficacy of sunitinib, VEGFRs, KIT, PDGFRs, and tyrosine kinase inhibitor (TKI) in twenty-eight patients (nineteen male/nine female) with stage III and IV thymomas (8) and TC (20) who already received other systemic treatments [21]. Sunitinib was administered as an initial daily dose of 50 mg in eleven patients, 37.5 mg in sixteen patients, and 25 mg in one patient. The mPFS was 3.7 months for the entire cohort, 5.4 months for patients with thymomas, and 3.3 months for patients with TC. The mOS for the whole population was 15.4 months (not reached for thymomas and 12.3 months for TC). The total objective response rate (ORR) was 22.2%, 28.6% for thymomas, and 20% for TC. The incidence of grade 3–4 treatment-related AE was 28.6% and the most frequent were stomatitis, asthenia, diarrhea, and a decrease in the left ventricular ejection fraction [21].

Thomas et al. conducted a phase II trial evaluating the efficacy of sunitinib in thymomas and TC [22]. Forty-one patients (twenty-three male/eighteen female) were enrolled in this trial. There were 25 TC and 16 thymomas treated with at least one platinum-based chemotherapy, and the mFU was 17 months. Sunitinib was administered as 50 mg orally once daily for six-week cycles (4 weeks on/2 weeks off treatment). The mPFS was 7.2 months (3.4–15.2) for TC and 8.5 months (2.8–11.3) for thymomas. The mOS was 15.5 months for thymomas, whereas it was not reached for TC. Grade 3 and 4 treatment-related AE were observed in 28 (70%) patients. The more frequent among them were lymphocytopenia (20%), fatigue (20%), oral mucositis (20%), and a decrease in the left ventricular ejection fraction (13%) [22].

Another phase II trial (NCT01621568) also investigated sunitinib toxicity and efficacy in patients with advanced TET with at least one prior line of platinum-based chemotherapy [23]. Patients in Group 1 (thymoma and TC) were treated with sunitinib 50 mg/day, 4 weeks on, 2 weeks off (6-week cycle). Patients in Group 2 (TC) were treated with sunitinib 50 mg/day, 2 weeks on, 1 week off (3-week cycle). In Group 1, the mPFS was 8.5 months (2.8–11.3) and for TC 7.2 months (3.4–15.2). In Group 2, it was 5.0 months (2.7–5.5). In Group 1, ninety-five grade 3 AE and six grade 4 AE were reported, whereas, in Group 2, forty grade 3 and three grade 4 AE were reported [23]. 

In the context of this study, a modified dose of 50 mg once daily using a 2 weeks on/1 week off schedule in patients with progressive TC after at least one prior platinum-containing chemotherapy regimen was applied and the results were presented in 2017 [24]. Among thirteen evaluable patients, there was one (8%) with partial response (PR), eleven (85%) with stable disease (SD), and one (8%) with PD. After mFU of 16 months, the mPFS was 5 months and the mOS was 16 months. Grade 3 or 4 treatment-related AE occurring in more than 10% of patients included lymphocytopenia (40%), neutropenia and leucopenia (20% each), thrombocytopenia, and oral mucositis (13% each). Grade 3 decrease in LVEF was observed in one (7%) patient [24].

Another phase II trial (NCT02623127) evaluated the clinical efficacy and toxicity of sunitinib in Korean patients with metastatic TC after platinum-based chemotherapy [25]. The results were presented in 2018. The investigators enrolled twenty-five patients (nineteen male/six female) with histologically confirmed platinum-refractory TC. The patients received 50 mg of sunitinib on an alternative schedule (2 weeks of treatment followed by 1 week without treatment). Among twenty-three evaluable patients, five (21.7%) patients had a PR, and sixteen (69.6%) patients achieved SD. The disease control rate (PR + SD > 6 m) was 56.5%. The mPFS was 15.2 months. The most common grade 3/4 toxicity was thrombocytopenia (four patients, 16%) [25].

The recently published STYLE trial (NCT03449173) is a phase II study conducted to assess the activity of sunitinib in patients affected by advanced or recurrent B3 thymoma or TC progressing after at least one line of chemotherapy (including one platinum-based regimen) [26]. Twelve patients with B3 thymoma and thirty-two patients with TC were enrolled. In the thymoma group, the ORR was 0% (90% CI: 0.0–22.1%) and DCR was 91.7% (95% CI: 61.5–99.8%). The median PFS was 7.7 months (95% CI: 2.4–45.5) and mOS was 47.9 months (95% CI: 4.5 not reached). In the TC group, ORR was 21.4% (95% CI: 8.3–41.0%), DCR was 89.3% (95% CI: 71.8–97.7%), mPFS was 8.8 months (95% CI: 5.3–11.1), and mOS was 27.8 months (95% CI: 13.2–53.2). The incidence of AE was 91.7% in the thymoma group (Grade 3 or greater treatment-related AE 25%) and 93.5% (Grade 3 or greater treatment-related AE 51.6%) in the TC group [26].

The efficacy of sunitinib was also evaluated in a retrospective multicenter study involving 20 patients (10 male/10 female) with stage IV platinum-refractory thymomas (8) and TC (12) [27]. The mPFS of the whole cohort was 7.3 months (4.5–10.3), 7.3 months for thymomas, and 6.8 months for TC. The ORR was 31.6% (12.5–56.5%). The incidence of grade 3–4 treatment-related AE was 30% (asthenia and fatigue 10%) [27]. 

Ströbel et al. retrospectively evaluated four patients with metastatic TC refractory to conventional therapies who were treated with sunitinib [19]. Partial remission (lasting 2 to >18 months) was achieved in three patients and stable disease with excellent metabolic response in another one. The OS ranged from 4 to >40 months. The toxicity profile of sunitinib was acceptable [19].

The multicenter Japanese phase II REMORA trial investigated the efficacy of lenvatinib, which is a multi-targeted inhibitor of VEGFR, FGFR, RET, c-Kit, and other kinases [28]. Forty-two patients (twenty-nine males/thirteen females) with TC (the majority were stage IVA and IVB) previously treated with at least one platinum-based chemotherapy were included. The mFU duration was 15.5 months. The mPFS was 9.3 months (7.7–13.9), whereas the mOS was not reached (16.1 not reached). On the contrary, the ORR was 38% (25.6–52%). The most frequent grade 3–4 treatment-related AE was hypertension (64%). Lenvatinib is a promising treatment approach because its clinical activity, with an ORR of 38%, is the highest rate documented to date in TET that progressed after first-line chemotherapy [28]. However, it is unknown whether its results could be reproduced in non-Asian populations. 

New small-molecule TKI with anti-angiogenic action are the subject of investigation. Anlotinib was effective in treating a patient with refractory TC who had received numerous lines of chemotherapy and anti-angiogenic therapy with another multi-target TKI, apatinib, according to a recent case report [29]. Over 23 months of PFS and six years of OS have been achieved. The AE were less severe and acceptable as compared to apatinib, and the patient’s quality of life ameliorated.

A phase II trial evaluated the efficacy of apatinib in 25 patients with stage IV TET (10 thymomas and 15 TC) [30]. The ORR and DCR in ten patients with T were 70% (95% CI 35–93%) and 100% (95% CI 69–100%), respectively. The ORR and DCR in 15 patients with TC were 20% (95% CI 4–48%) and 73% (95% CI 45–92%), respectively. The mPFS was 9.0 (95% CI 5.4–12.6) months in the entire population, 9.5 (95% CI 8.6–10.4) months in patients with thymomas, and 6.1 (95% CI 2.6–9.6) months in 15 patients with TC. The mOS was 24.0 (95% CI 8.2–39.8) months in all 25 patients, 22.4 (95% CI 6.4–38.4) months in 10 patients with thymomas, and 24.0 (95% CI 16.1 not evaluable) months in 15 patients with TC. The most common grade 3 treatment-related AE included hypertension (32%), hand–foot syndrome (20%), proteinuria (12%), lymphocytopenia (12%), fatigue (8%), nausea (4%), vomiting (4%), oral mucositis (4%), increased levels of gamma-glutamyltransferase (4%), and neutrophilic granuloacytopenia (4%). No grade 4 or 5 AE were reported [30].

Guan et al. retrospectively collected data on clinical progress after first-line chemotherapy in TC patients who were treated with small-molecule anti-angiogenic agents [31]. Of the seventeen patients enrolled, thirteen (76.5%) received apatinib and four (23.5%) anlotinib monotherapy with an ORR of 23.5%. Eleven (64.7%) patients had SD. The mFU period was 46 months. The mPFS and mOS were 7.9 months (95% CI, 6.5–9.3) and 47 months (95% CI, 35.4–58.6), respectively. In the 13 patients receiving apatinib, the mPFS was 7.0 months (95% CI, 5.0–9.0), compared with 8 months (95% CI, 2.7–13.3 months) for patients in the anlotinib group (*p* = 0.945). The most common grade 3 AE were hypertension (23.1%), followed by proteinuria and hand–foot syndrome (15.4%). There were no grade 4 AE; however, eight patients (47.1%) required mid-course discontinuation [31]. 

Li et al. retrospectively assessed the clinical efficacy and safety of anlotinib in previously treated TET patients [32]. Twenty-two patients were enrolled, including eighteen cases of TC and four cases of thymoma. Fourteen patients (63.6%) received anlotinib monotherapy and eight patients (36.4%) received anlotinib combination therapy. The anlotinib combination group was treated with anlotinib combined with either immunotherapy (camrelizumab, sintilimab, or tislelizumab) or chemotherapy (gemcitabine plus cis-platinum/tegafur, docetaxel plus vinorelbine, and epirubicin). The ORR was 9.1% in the overall cohort. Two patients with TC achieved PR, and the ORR for TC was 11.1% (9.1% for anlotinib monotherapy and 14.3% for anlotinib combination therapy). The mPFS in the overall population was 12 months (14 months for thymomas and 9 months for TC), and the mOS was 24 months (not reached for thymomas and 24 months for TC). The incidence of AE was 50%, and the incidence of grades III and IV AE was 9% (arterial thromboembolism) [32].

Yudong et al. reported a case of advanced TC harboring EGFR exon 20 insertion in which a response was achieved using apatinib after multi-line chemotherapy and radiotherapy [33]. The patient achieved PR with a 31% reduction in tumor size and a PFS of 10 months. Grade 3 hand–foot syndrome was recorded.

Lucitanib is a potent oral selective inhibitor of the tyrosine kinase activity of FGFR1-3, VEGFR1-3, and PDGFR α/β [34]. In a phase I trial, three patients had B-type thymoma and twelve had TC. Twelve patients (80%) were treated with 12.5 mg on a daily basis. The other three patients received 5, 15, and 20 mg, respectively. The patients had received a median of two previous anti-cancer treatments [range: 0–6]. The median duration of treatment with lucitanib was seven cycles [range 2–44]. Two patients had confirmed PR lasting at least 7 months, and ten patients had SD, with 6 of them lasting at least 6 months. The most common adverse events related to lucitanib in this population (regardless of grade and dose) were hypertension (80%), hypothyroidism (53%), proteinuria (53%), and diarrhea (40%). The authors conclude that lucitanib may be clinically active in patients with advanced TET, and these findings warrant additional research in focused trials [34].

Bedano et al. conducted a phase II trial that studied the efficacy and safety of the combination of erlotinib (EGFR inhibitor) and bevacizumab (VEGFR inhibitor) on pretreated patients with progressive TET (eleven thymomas and seven TC) [17]. Eighteen patients were included (eight male/ten female). Among them, none achieved a complete response, eleven patients (60%) achieved SD, and seven (40%) had PD. No grade 4 toxicities or deaths were reported. The most frequent grade 3 AE were acneform rash, dyspnea, fatigue, pericardial tamponade, and aortic insufficiency. The median survival time has not been reached. The authors concluded that, despite having a well-tolerated side effect profile, the combination of erlotinib and bevacizumab shows only little action in thymic malignancies. Although an effect of the drugs cannot be excluded, the high occurrence of SD most likely reflects the innate tumor biology [17]. 

## 3. Discussion

Typically, angiogenesis occurs under physiological circumstances, such as those that arise during embryonic development, wound healing, bone repair, and regeneration [2]. Additionally, specific pathological entities such as tumors, immune system disorders, inflammatory conditions, and hematological diseases can cause it. A key factor in the development and progression of disease is pathological angiogenesis. It plays a crucial part in the development of tumors, primarily by delivering nutrients and oxygen to tumor cells, aiding in their spread, and creating an immunosuppressive tumor microenvironment that results in tumor evolution [1,3,4,35,36]. Recent studies suggest that disruption of the equilibrium between activine A and its naturally occurring inhibitor, follistatin, is one of the pro-angiogenic pathogenic pathways in TET. High follistatin levels were observed in TC patients and were related to tumor microvessel density and advanced tumor stage [35].

Because every tissue has unique properties and vascular characteristics that set separate organs apart, it is believed that different tumor types use different genetic processes to generate blood circulation, depending on their stage of development and environment [9]. The neoplastic cell can release sequestered growth factors or their receptors from the extracellular matrix, or it can attract inflammatory cells like mast cells and macrophages, which are both abundant sources of cytokines and angiogenetic factors. A rich source of angiogenic agents, platelets are also frequently found in higher concentrations in malignant diseases. Tumor endothelium or epithelium can activate platelets [9,37]. The first soluble angiogenic factor, tumor angiogenesis factor (TAF), was identified by Folkman et al. in 1971 [38].

The VEGF family, which presently consists of placental growth factor and VEGF-A to VEGF-D, is involved in a variety of human tumor types [39]. These bind variably to three high-affinity endothelial-cell tyrosine kinase receptors: flt-1 (VEGFR1), KDR (VEGFR2), and flt-4 (VEGFR3) [9]. This complicated cascade allows the VEGFs to have many effects, such as increasing vascular permeability (which results in increasing tumor stroma development), endothelial cell proliferation, and tube formation.

Many tumors of the lung, brain, gastrointestinal system, and urogenital tract express VEGF-A at high levels.

The correlation between expression and microvessel density and prognosis demonstrates the significance of VEGF-A in human malignant illness. The other VEGFs’ function in human disease is still being investigated, though [9,39]. It is now understood that a tumor’s net angiogenic activity is determined by the ratio of angiogenic stimulators to inhibitors. Thus, angiogenesis may be caused by the overexpression of favorable factors and/or the suppression of a number of naturally occurring inhibitors [9].

Numerous distinct cells create angiogenetic growth factors, which have a close role in both tumor angiogenesis and development. Angiogenic growth factors, including VEGF, FGF, and angiopoietin, are crucial for the process of angiogenesis [40]. These growth factors are produced by various cell types and include a diverse range of proteins in addition to VEGF and FGF: platelet-derived growth factor, tumor necrosis factor, insulin-like growth factor-1, transforming growth factor, angiogenin, hepatocyte growth factor, placental growth factor, and several others. The FGF and VEGF families of angiogenetic growth factors have been studied the most out of all of those that have been identified.

### 3.1. Fibroblast Growth Factor

These molecules are essential to the process of angiogenesis because they promote the growth of both fibroblasts and endothelial cells. They are also involved in at least three of the four stages of wound healing: inflammation, repair, and regeneration. Tumor formation and progression are among the other significant roles played by FGFs.

The two isoforms that have been studied the most are FGF-1 and FGF-2 [40]. 

### 3.2. Vascular Endothelial Growth Factor

There are currently at least five members of the VEGF family, and three VEGF receptors (VEGFR) mediate their activities. Transmembrane receptor tyrosine kinases (RTKs) allow these receptors to communicate with the interior of cells [9,40,41].

### 3.3. VEGF Receptors

In humans, VEGFR-1 and VEGFR-2, two high-affinity membrane-spanning receptors, mediate the actions of VEGF on endothelial cells [9,40,41].

### 3.4. Side Effects in Anti-Angiogenic Therapy

Angiogenesis inhibitors have been linked to possible disruptions of numerous physiological functions, including blood pressure, kidney function, wound healing, fetal development, reproduction, and an increased risk of thrombi in the arteries that could cause a heart attack or stroke. For instance, one of the most common adverse effects of systemic VEGF signaling suppression is hypertension, which is also one of the easiest to control when using prescription anti-hypertensive drugs. When VEGF signaling is inhibited in cancer treatment, the resulting reduction in VEGF levels causes endothelial dysfunction and ultimately leads to hypertension [41]. 

### 3.5. Categories of Angiogenesis Inhibitors

The development of new blood vessels can be prevented by angiogenesis inhibitors, which would stop tumor growth but not completely eradicate it. Consequently, anti-angiogenesis monotherapies do not work as well in humans as was anticipated. Combinatorial therapies using traditional chemotherapeutic medications are therefore necessary [2,42]. There are two primary categories of angiogenesis inhibitors: (i) direct inhibitors, which act on endothelial cells inside the expanding vasculature, and (ii) indirect inhibitors, which act on tumor cells or other stromal cells linked with tumor growth [41]. Direct inhibitors of angiogenesis include angiostatin, endostatin, arrestin, canstatin, and tumstatin. They impede the proliferation and migration of vascular endothelial cells in response to certain activators of angiogenesis, such as VEGF, bFGF, IL-8, and PDGF [43,44]. Endogenous inhibitors that directly target those signaling pathways were believed to have the lowest potential to develop resistance to drugs because they attack genetically stable endothelium cells rather than unstable tumor cells that are mutating. However, in randomized phase III trials, endostatin has not yet been shown to benefit patients in any way, and, in phase II trials, it has not even produced moderate action [43,45].

On the other hand, pro-angiogenic proteins like EGFR will not express or function when indirect angiogenesis inhibitors are used [44]. Gefitinib, an EGFR TKI, has been used as a treatment in different human cancers (colon, ovary, and breast) [46].

In general, small-molecule tyrosine kinase inhibitors (TKIs), VEGF decoy receptors, and monoclonal antibodies are the three main groups of medicines that target VEGF that have been produced [47].

Currently, these drugs are being studied or used in clinical settings either alone or in conjunction with radiation or cytotoxic chemotherapy.

#### 3.5.1. Anti-VEGF Monoclonal Antibodies

Bevacizumab is a recombinant humanized monoclonal antibody against VEGF and inhibits VEGF-A produced by tumor cells, thus hindering the formation of new blood vessels and ultimately causing tumor starvation and growth suppression [48]. However, combining bevacizumab with chemotherapy has been shown to exacerbate its negative effects. As already mentioned, the administration of bevacizumab is validated in randomized phase III trials for the treatment of colorectal cancer in conjunction with chemotherapy. FDA approval was attributed to bevacizumab for the treatment of advanced non-small-cell lung cancer of non-squamous histological type [49,50]. Glioblastoma, renal cell carcinoma, and metastatic HER2-negative breast cancer are other cancer types in which the administration of bevacizumab is assessed for treatment [51,52,53,54].

A systematic review provided data about the adverse events related to the use of bevacizumab in colorectal cancer patients [55]. The four categories of adverse effects include hematological, cardiovascular, gastrointestinal, and other. Cardiovascular complications include coronary artery disease, myocardial infarction, arrhythmias (atrial fibrillation and atrioventricular block), arterial hypertension, and thromboembolic events, even though the latter occur less frequently. The most important gastrointestinal complication is perforation of the digestive tube. When bevacizumab is added to chemotherapy, bleeding occurs more frequently. Neutropenia, febrile neutropenia, anemia, and thrombocytopenia are among the reported hematological complications.

#### 3.5.2. VEGF Decoy Receptor

Aflibercept inhibits VEGF-A, VEGFB, and PIGF2 and has a stronger affinity for VEGF-A than BEV. Phase I–II trials encouraged more research on the use of aflibercept in conjunction with chemotherapy for a variety of cancer types [47]. Adverse events such as diarrhea, asthenia, hypertension, proteinuria, infections, and neutropenia are related to the administration of aflibercept in patients presenting metastatic colorectal cancer [56].

#### 3.5.3. Small-Molecule Inhibitors

The biochemical function of the downstream VEGFR-mediated signaling tyrosine kinases can be potently and selectively inhibited by small-molecule tyrosine kinase inhibitors (TKIs), particularly the multi-target kinase inhibitors. These multi-targeted oral TKIs are thought to have a wide range of inhibitory effects on tumor angiogenesis and growth. They target angiogenesis pathways, such as VEGFR, PDGFR, FGFR, c-KIT, FLT-3, etc. [57,58,59,60].

Imatinib is a selective inhibitor of Bcr/Abl, and it is approved for the treatment of hematological malignancies and gastrointestinal stromal tumors.

Sorafenib targets VEGFR-2 and -3, PDGFR-b, Flt-3, and c-Kit and is used in the treatment of hepatocellular carcinoma and renal cell carcinoma. Toxicities include diarrhea, rash, nausea, cardiac ischemia, or infarction [47]

Sunitinib is a multi-TKI that targets VEGFR-1–3, PDGFR, Flt-3, and c-Kit79.

It was first used for the treatment of GIST after failure of treatment with imatinib, and it was later approved for metastatic renal cell carcinoma [47].

Axitinib is a potent second-generation inhibitor of VEGF-1, 2, and 3. In contrast to first-generation inhibitors, it has better VEGF-specific selectivity and does not block PDGF, b-RAF, FLT-3, and KITor other off targets. It is mostly used in the case of renal cell carcinoma. Toxicities include diarrhea, hypertension, fatigue, nausea, and dysphonia [47].

Lenvatinib is a multiple-receptor TKI of VEGFR1-3, FGFR1-4, KIT (tyrosine-protein kinase KIT or mast/stem cell growth factor receptor), platelet-derived growth factor receptor a (PDGFRa), and RET [47].

Anlotinib is a multiple-kinase inhibitor that has demonstrated effectiveness against different cancer types. Its use has recently gained interest against certain types of thyroid malignancies [61].

There is some evidence that angiogenesis plays a significant part in thymic epithelial malignancies. VEGF is overexpressed in these cancers, and microvessel density and VEGF expression are related to invasiveness and stage [36,62,63]. Patients with TC have been found to have higher serum levels of VEGF and b-FGF [31]. Additionally, in thymic epithelial cells, PDGF and PDGFR are overexpressed [64]. VEGF-, KIT-, or PDGF-targeting medications may be effective in treating TET according to anecdotal findings [18]. Three of the four patients with TC showed responses to sunitinib and multiple-receptor tyrosine kinase activation according to research by Strobel et al. [19].

Lattanzio et al. used immunohistochemistry to assess the expression of possible molecular targets of anti-angiogenic therapy, such as VEGFA, VEGFC, VEGFD, VEGFR1, VEGFR2, VEGFR3, and PDGFRβ, in a Tissue Micro Arrays series of 200 TET [3]. B3 thymomas and TC expressed significantly higher levels of both VEGFA and VEGFC compared to A, AB, and B1 thymomas. Additionally, compared to stage I and stage II tumors, stage IV tumors displayed a larger proportion of VEGFA- and VEGFC-positive cells [3].

Lenvatinib, an anti-angiogenic TKI investigated in phase II trials, demonstrated efficacy in TC, with a remarkable ORR. Also, sunitinib showed a high response rate and may therefore be a good option. Based on comparison between trials, which should be conducted cautiously, it has been considered that lenvatinib attained a higher response rate in comparison to other drugs, with a better toxicity profile. Lenvatinib may be more effective because it acts by inhibiting different tyrosine kinase receptors, such as VEGFR2, and different pharmacodynamic features could possibly be involved [28].

In clinical trials, sunitinib has been reported to be beneficial against metastatic clear-cell renal carcinoma [65], gastrointestinal stromal tumors [66], and advanced pancreatic neuroendocrine tumors [67]. The efficacy of sunitinib in TET was encouraging, and, as a result, sunitinib has been recommended as an option in the European Society of Medical Oncology (ESMO) guidelines [68].

Disappointingly, the investigators of the Resound trial were not able to identify a subset of patients potentially benefiting from regorafenib. In fact, no difference was observed in terms of DCR, PFS, or OS stratifying for age, histology, sex, response to the previous line, and line of therapy [20].

Grade 3 and 4 treatment-related AE, dose reduction, and definitive drug interruption were observed in ten (52.6%), nine (47.4%), and three (15.8%) of nineteen patients treated with regorafenib, respectively. These results are similar to those achieved by sunitinib (grade 3–4 treatment-related AEs 70%; dose reduction 65%; definitive drug interruption 18.5%) and lenvatinib (grade 3–4 treatment-related AEs 64%; dose reduction 100%; definitive drug interruption 17%) [21,22,28]. No grade 5 treatment-related AEs were observed with regorafenib [20].

For patients with TET, new small-molecule TKI with anti-angiogenic action are being investigated. Anlotinib is a new oral TKI with a broad therapeutic index that can effectively inhibit VEGFR, PDGFR, FGFR, and c-kit. Anlotinib exhibits significant VEGF receptor VEGFR2 and VEGFR3 selectivity [31,69,70]. However, there are mainly retrospective series reporting on their efficacy in the treatment of TET. On the other hand, a recent phase II trial assessing apatinib in stage IV TET [30] yielded encouraging results.

RELEVENT is a multicentric open-label phase 2 study (NCT03921671) [60]. Patients with TET of any histological type will be enrolled in this trial. Its objective is to evaluate the activity and safety of the combination of ramucirumab (10 mg/kg) plus carboplatin (AUC 5) and paclitaxel (200 mg/m^2^) in patients with relapsed and/or metastatic TC/thymoma B3 after first-line treatment [60]. Ramucirumab is a fully human monoclonal antibody (IgG1) that acts as a direct VEGFR2 antagonist that binds with high affinity to the extracellular domain of VEGFR2 and blocks the binding of natural VEGFR ligands (VEGF-A, VEGF-C, and VEGF-D) [71].

Pro-angiogenic factors cause dysregulated pathological tumor vasculature’s progression. Consequently, the tumor microenvironment is characterized by hypoxia, acidity, patchy hypoperfusion, and high interstitial fluid pressure. Tumor vasculature is also characterized by structural abnormalities [4]. All these variables may have a major impact on the immunotherapeutic response and may have an impact on immune cell survival, infiltration, proliferation, and function, suppressing the tumor microenvironment. Immune cell infiltration and function in the tumor microenvironment play a significant role in immunotherapy efficacy. The ability of anti-angiogenesis to normalize blood vessels in tumors was subsequently identified through ongoing research, and this offered the rationale for combining it with a variety of anti-tumor medicines [4]. The use of immunotherapy and vascular normalization therapy as standalone treatments for tumors has recently become increasingly important, although each faces a number of obstacles. Numerous studies have uncovered intricate regulatory relationships between an immunosuppressive tumor microenvironment and aberrant angiogenesis, and they have also confirmed the therapeutic efficacy of immunotherapy and anti-angiogenesis treatment when used in combination [1,4,72]. The importance and efficiency of this combined strategy have also been supported by an increasing number of clinical trials. Immunotherapy is probably more successful when combined with vascular normalization therapy, which can also decrease adverse effects and extend patients’ survival [1]. Pembrolizumab in conjunction with sunitinib or lenvatinib in patients with TC is being studied in two phase II trials (NCT03463460 and NCT04710628) [73,74]. In a different phase I/II study, patients with thoracic malignancies, including TC, will be administered oral VEGFR/PDGFR TKI vorolanib in combination with nivolumab (NCT03583086) [75]. The ongoing trials registered in clinicaltrials.gov are demonstrated in Table 2.

Treatment with the combination of immune checkpoint inhibitors (ICI) and small-molecule anti-angiogenic drugs showed encouraging results in some solid tumors [76,77]. Therefore, it is necessary to continue research on innovative combinations of ICIs and small-molecule anti-angiogenic medications in patients with recurrent TC.

## 4. Materials and Methods

This article was designed according to the recent recommendations on the quality assessment of narrative review articles [78]. PubMed research was conducted using the terms [anti-angiogenics] AND [thymic epithelial tumors] OR [thymomas] OR [thymic carcinoma] AND [angiogenesis inhibitors] AND [thymic epithelial tumors] OR [thymomas]. Papers concerning pediatric cases and non-English literature papers were excluded. Papers were chosen based on relevance because the current study is not a systematic review. The references of selected papers were sought in order to find other pertinent articles. There was no restriction concerning the publication date.

## 5. Conclusions

This review pointed out that anti-angiogenic agents may be useful in the treatment of TET, which are not amenable to curative treatment, especially when traditional chemotherapy schemes fail. Their toxicity profile seems to be acceptable. However, angiogenesis inhibitors do not appear to yield a high response rate on either thymomas or TC, although multikinase inhibitors may have some effect on TC. The current evidence suggests that the most active agent is lenvatinib, as demonstrated in the REMORA phase II trial, whereas sunitinib could be proposed as an acceptable second-line therapy for TC. Further research concerning the combination of ICI with anti-angiogenic drugs is warranted; however, it has to be taken into account that evidence is not expected to be robust as it will not be based on large-scale randomized controlled trials because the rarity and histological heterogeneity of TET hinder conducting this kind of research.

## Figures and Tables

**Table 1:** Phase II trials and prospective studies with published results evaluating the efficacy of anti-angiogenic drugs in the treatment of thymic epithelial tumors.

Author/Year	Type of Study	Molecule	Patients Enrolled	Histology	mPFS (Months)	mOS (Months)	ORR	DCR	Grade 3–4 AE
Perrino 2022 [20] (Resound trial)	Phase II	Regorafenib	19	11 thymomas/8 TC	9.6 (95% CI, 3.6–12.8%)	33.8 (95% CI, 10.2% not reached)	NR	78.9% (95% CI, 54.4–94.0%; N = 15)	52.6%
Remon 2016 [21]	Prospective cohort	Sunitinib	28	8 thymomas/20 TC	Overall population 3.7 (5.4 thymomas, 3.3 TC)	Overall population 15.4 (not reached thymomas, 12.3 TC)	Overall 22.2%, thymomas 28.6%, TC 20%	63% (86% for thymomas, and 55% for TC)	28.6%
Thomas 2015 [22]	Phase II	Sunitinib	41	16 thymomas/25 TC	TC: 7.2 (3.4–15.2), thymoma: 8.5 (2.8–11.3)	TC: not reached, thymoma: 15.5 (12.6 undefined)	NR	TC: 91% (95% CI 72.0–98.9), thymomas: 81%, 54·4–96·0	70%
Rajan 2023 [24]	Phase II	Sunitinib	56	Group 1 (Thymoma and TC): 41 Group 2 (TC only): 15	Group 1: 8.5 (2.8–11.3) and for TC 7.2 (3.4–15.2). Group 2: 5.0 (2.7–5.5)	NR	NR	NR	Group 1: 95 grade 3 AE and 6 grade 4 AE Group 2: 40 grade 3 and 3 grade 4 AE
Proto 2023 (STYLE trial) [26]	Phase II	Sunitinib	44	12 B3 Thymomas/32 TC	Thymomas: 7.7 months (95% CI: 2.4–45.5), TC: 8.8 months (95% CI: 5.3–11.1)	Thymomas: 47.9 months (95% CI: 4.5 not reached), TC: 27.8 months (95% CI: 13.2–53.2)	Thymomas: 0% (90% CI: 0.0–22.1%), TC: 21.4% (95% CI: 8.3–41.0%)	Thymomas: 91.7% (95% CI: 61.5–99.8%), TC: 89.3% (95% CI: 71.8–97.7%)	Thymoma group: 25% TC group: 51.6%
Song 2022 [30]	Phase II	Apatinib	25	10 Thymomas/15 TC	Overall population: 9.0 (95% CI 5.4–12.6), Thymomas: 9.5 (95% CI 8.6–10.4), TC: 6.1 (95% CI 2.6–9.6)	Overall population: 24.0 (95% CI 8.2–39.8), Thymomas: 22.4 (95% CI 6.4–38.4), TC: 24.0 (95% CI 16.1–∞)	Thymomas: 70% (95% CI 35–93%), TC: 20% (95% CI 4–48%)	Thymomas: 100% (95% CI 69–100%), TC: 73% (95% CI 45–92%)	Grade 3 60%. No grade 4 AE
Sato 2020 (REMORA trial) [28]	Phase II	Lenvatinib	42	TC	9.3 (7.7–13.9)	Not reached (16.1 not reached)	38% (25.6–52%)	95% (83.8–99.4)	Hypertension 64%, palmar–plantar erythrodysaesthesia syndrome (7%)
Bedano 2008 [17]	Phase II	Erlotinib/bevacizumab	18	11 Thymoma, 7 TC	NR	Not reached	0%	61%	38.8%

TC: thymic carcinoma; mPFS: median progression-free survival; mOS: median overall survival; ORR: objective response rate; DCR: disease control rate; AE: adverse events; NR: not reported.

**Table 2:** List of ongoing trials (clinicaltrials.gov).

NCT Number	Status	Histology	Drugs Assessed	Study Type	Number of Patients Enrolled (or Estimated)
NCT03921671 (RELEVENT trial)	Unknown	B3 Thymomas/TC	ramucirumab + carboplatin and paclitaxel	Phase 2	60
NCT03463460	Recruiting	TC	pembrolizumab and sunitinib	Phase 2	40
NCT04710628	Recruiting	B3 Thymomas/TC	pembrolizumab and lenvatinib	Phase 2	43
NCT03583086	Active not recruiting	TC	vorolanib plus nivolumab	Phase 1/2	88 (overall population of different thoracic tumors)
NCT01306045	Active not recruiting	TC	AZD6244, MK-2206, erlotinib, sunitinib, lapatinib (according to molecular profiling)	Phase 2	647 (overall population of different thoracic tumors)

TC: thymic carcinoma.

## Data Availability

No new data were created or analyzed in this study.
